# Ciprofloxacin-Induced Acute Delirium in a Young Female

**DOI:** 10.7759/cureus.25182

**Published:** 2022-05-21

**Authors:** Zaland A Yousafzai, Qazi Kamran Amin, Wajeeha Qayyum, Azhar Saeed, Nouman Anthony

**Affiliations:** 1 General Medicine, Rehman Medical Institute, Peshawar, PAK; 2 Gastroenterology, Rehman Medical Institute, Peshawar, PAK; 3 General Medicine, Rehman Medical Institue, Peshawar, PAK

**Keywords:** adverse side effect, antibiotics therapy, side effects, ciprofloxacin, delirium

## Abstract

Ciprofloxacin is a commonly used drug in our setup. Neurological disturbances are rare side effects of its use and are reported in older adults, patients with comorbidities, or patients with a background of psychiatric illness and antipsychotic drug use. We report the case of a 21-year-old female who developed delirium after taking ciprofloxacin for a urinary tract infection. She underwent extensive workup and her final diagnosis was based on the exclusion of other causes. Her symptoms completely settled within three weeks of ciprofloxacin discontinuation.

## Introduction

Ciprofloxacin is a second-generation fluoroquinolone mostly used against aerobic Gram-negative, some Gram-positive, and atypical intracellular microorganisms. GI disturbances, hypersensitivity reactions, photosensitivity, and tendinopathies are all common side effects of ciprofloxacin use. Rare side effects can include neurological manifestations, psychosis, and altered mentation [1,2].

## Case presentation

A 21-year-old female with no previous comorbidities presented to the emergency room in an acute confusional state and had episodes of agitation and restlessness for the last two days, along with sleep disturbance. Her vital signs were normal, with a blood pressure of 125/85 mmHg, a heart rate of 76/min, a respiratory rate of 14 min, oxygen saturation of 99% on room air, and a temperature of 98.4 °F. Her general physical exam and neurological exam were normal. She was restless and did not respond to verbal commands.

Complete blood count, C-reactive protein level, urine routine examination, serum electrolytes, blood sugar, renal function tests, and urine for toxicology screening were normal except for serum alanine aminotransferase (ALT) level, which was 90 IU. She was evaluated for organic neurological disorders. A CT scan and MRI brain with contrast were normal, along with cerebrospinal fluid (CSF) studies including CSF routine examination, herpes simplex virus (HSV) polymerase chain reaction (PCR), CSF culture, and CSF testing for autoimmune encephalitis, all of which revealed no abnormality. The electroencephalogram (EEG) was normal. A repeat liver function profile showed an increase in serum ALT level of 537 IU 4 days later. Her viral serology, complete liver profile, serum ammonia, and CT scan liver dynamic were unremarkable. Over the period of one week, her mental state worsened with increased restlessness, agitation, and aggression. A psychiatric evaluation was carried out to explore any past history of psychiatric symptoms or personality disturbance. Nothing significant was found to support this episode as a feature of psychiatric illness.

Delirium was labeled using the Confusion Assessment Method (CAM) according to which features of acute onset, fluctuating course, inattention, disorganized thinking, and altered level of consciousness were present. As all possible causes of delirium were ruled out, a detailed medical history was retaken, which revealed a history of urinary tract infection one week before the onset of her confusion. She was prescribed ciprofloxacin 500 mg BD by a general practitioner, which caused a generalized body rash that subsided after the discontinuation of the drug. Her confusional status started at the same time. Based on this history and excluding all possible causes of delirium, a diagnosis of ciprofloxacin-induced delirium was made. Ciprofloxacin was discontinued immediately, and for symptomatic relief of her agitation and aggression, haloperidol 2.5 mg IM was used on a PRN basis. Her symptoms started to settle within a week, along with a declining trend in her serum ALT levels. After three weeks of a follow-up visit, the patient’s mental status had returned to her baseline status, and her ALT levels had also normalized.

## Discussion

Ciprofloxacin is used as one of the most common antibiotics for urinary tract infections, upper respiratory tract infections, and other common infections in our system. In the literature on neuropsychiatric side effects of Ciprofloxacin, they are rare but have been reported. The neuropsychiatric side effects of Ciprofloxacin can be attributed to its antagonistic action on inhibitory GABA (gamma-aminobutyric acid) pathways and stimulation of adenosine and NMDA (N-methyl-D-aspartate) excitatory pathways [3]. They are reported in older adult patients or in those with comorbid states. A study from 2018 involving 631 hospitalized patients receiving fluoroquinolones found that 3.6% of ciprofloxacin-treated patients developed psychosis or delirium [4]. Concurrent use of antipsychotic drugs and increasing age were two risk factors responsible for delirium. Few reports of ciprofloxacin-induced delirium were reported in patients having a history of chronic kidney disease and chronic obstructive pulmonary disease (COPD) [5,6]. Whereas in our case, the patient had no previous history of any comorbidity, nor was she using any antipsychotic drugs. She was young and healthy prior to the onset of delirium.

Moreover, in our case, the patient developed multiple side effects secondary to ciprofloxacin, including the appearance of a generalized body rash, a rise in serum ALT levels, and delirium. In most cases, recovery was rapid after discontinuation of therapy, but in our patient, recovery was prolonged and it took three weeks for the patient to return to her baseline status.

## Conclusions

Ciprofloxacin is being used as one of the most common antibiotics for urinary tract infections and upper respiratory tract infections in our setup. Clinicians should be aware of the possible neurological side effects of Ciprofloxacin before prescribing it. Furthermore, the unjudicial use of antibiotics should be discouraged as it can lead to extensive workups and delayed diagnosis in patients developing rare side effects, as in our case.
